# High speed coding for velocity by archerfish retinal ganglion cells

**DOI:** 10.1186/1471-2202-13-69

**Published:** 2012-06-18

**Authors:** Viola Kretschmer, Friedrich Kretschmer, Malte T Ahlers, Josef Ammermüller

**Affiliations:** 1 Department of Biology and Environmental Sciences, Neurobiology, University of Oldenburg, 26111, Oldenburg, Germany; 2 Department of Biology and Environmental Sciences, Computational Neuroscience, University of Oldenburg, 26111, Oldenburg, Germany

**Keywords:** Retina, Ganglion cells, Rate coding, Latency coding

## Abstract

**Background:**

Archerfish show very short behavioural latencies in response to falling prey. This raises the question, which response parameters of retinal ganglion cells to moving stimuli are best suited for fast coding of stimulus speed and direction.

**Results:**

We compared stimulus reconstruction quality based on the ganglion cell response parameters latency, first interspike interval, and rate. For stimulus reconstruction of moving stimuli using latency was superior to using the other stimulus parameters. This was true for absolute latency, with respect to stimulus onset, as well as for relative latency, with respect to population response onset. Iteratively increasing the number of cells used for reconstruction decreased the calculated error close to zero.

**Conclusions:**

Latency is the fastest response parameter available to the brain. Therefore, latency coding is best suited for high speed coding of moving objects. The quantitative data of this study are in good accordance with previously published behavioural response latencies.

## Background

Archerfish have the ability to down aerial prey by shooting precisely aimed jets of water according to size, distance and weight of the prey [1]. As archerfish are swarm fish and compete for food, they need to calculate the impact point of shot down prey on the water surface, turn towards it, and match the swimming speed in order to be the first reaching the impact point. This calculation has to take place in the first few milliseconds of the prey falling and includes the trajectory of the prey, as well as the needed turning angle and the distance to the estimated point of impact. Behavioural experiments show latencies down to 40 ms for the first motor reaction after presentation of a falling prey [2]. Fast turning and acceleration might be achieved by the archerfish’s C-start escape network, which involves large, reticulospinal neurons associated with the Mauthner cells, and which shows fast responses to visual stimuli for life saving purposes [3-5].

Throughout different species retinal ganglion cells are well known to process motion-related information like speed [6,7] or direction [8-10]. In turtle and rabbit retina, for example, the firing rates of direction sensitive ganglion cells depend on a combination of both direction and speed of a movement [11,12]. Many of these studies focused on changes in the firing rate [12-14], where the number of spikes induced in a certain period of time conveys the necessary information. More recently, spike timing [15,16] with temporally precisely fired spikes increasingly attracted attention for understanding the neural code [17-20].

As archerfish show very short behavioural latencies to moving stimuli they are ideally suited to compare rate coding versus latency coding. In this study we, therefore, analysed archerfish ganglion cell responses to stimuli moving with various velocities and tested stimulus reconstruction quality using a maximum likelihood estimation procedure. We found that velocity reconstruction was superior using first spike latency in the reconstruction procedure, compared to first inter-spike interval and to spike rate.

## Results

**Figure 1:**
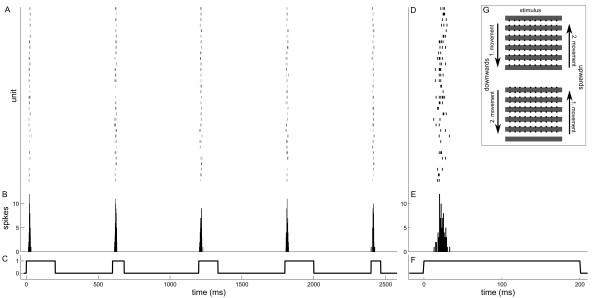
**Responses to different velocities.** (**A**) Example raster plot showing the activity of the 32 recorded ganglion cells from experiment 2 during 2500ms of movement stimulation with various velocities (2 m/s, 5 m/s, 3 m/s, 2 m/s, 6 m/s). The responses show a precise timing with short latencies to stimulus onset. (**B**) Summed population PSTH of all units over the single stimuli of all units from (**A**). (**C**) Stimulus trace. Note the different stimulus durations due to varying velocities. In (**D**) and (**E**) the responses and PSTH to the first stimulus (2 m/s) are shown at higher temporal resolution. Velocities are expressed as velocities of an external object at approximately 30cm distance from the eye (see Methods). (**G**) Stimulation pattern. A regular grating of 400μm wide black and white bars aligned with the electrode rows was constantly projected onto the retina. Stimulation started by moving the grating up or down. Movement stopped when the edges aligned with the next electrode row (movement 1). The following movement started into the same direction (movement 2) to achieve a grating reversal. Afterwards the movement direction also reversed.

The retina was stimulated with a black and white grating (see inset Figure 1 G). The grating constantly illuminated the retina, with the contrast borders aligned to the electrode rows of the multi-electrode array (MEA). Stimulation started by moving the grating up or down with one of 22 different, randomly chosen velocities. Stimulation paused when the contrast borders reached the neighbouring electrode rows (Figure 1 G). Movement direction changed after every second movement step. This ensured that the grating reversed in half of the stimuli.

Generally, the recorded ganglion cells showed precise spike timing with short latencies after movement onset when stimulated with the different velocities. This is depicted in the example shown in Figure 1. The raster plots show that all units elicited only a few spikes within a small time window (Figures 1 A & D). In this example spontaneous activity was completely absent, and at movement offset no responses were observed. The population PSTH of all units illustrates the sharp response peaks for all velocities (Figures 1 B & E).

### Comparison of stimulus reconstruction with latency, first inter-spike interval and rate

To see which response component conveys most information about the 22 velocities, we calculated latencies, the first inter spike interval (ISI) and the rate in a 100 ms time window after stimulus onset. In addition to the latencies calculated with respect to the known stimulus onset (absolute latencies) we also calculated the population responses to the various velocities and determined the latencies of the single ganglion cell responses with reference to these population response onsets. This will be termed relative latencies in the following.

**Figure 2:**
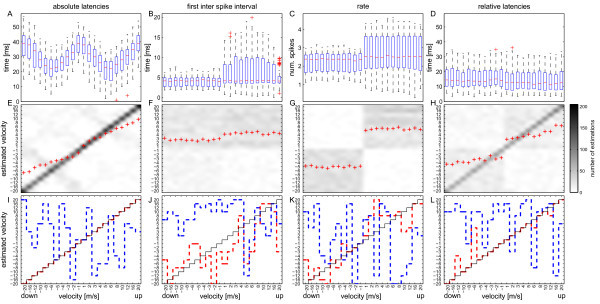
**Comparison of absolute latency, first inter spike interval, rate and relative latency.** (**A**) The tuning curve for absolute latencies of the whole population over all stimuli showed clear velocity dependence. The median of the fastest responses was 22ms at 6m/s for both directions. (**B**) In the tuning curves of the whole population for the fist inter spike intervals and (**C**) spike rate no velocity dependence was observed. The response variability to up- and downward movements, however, differed. The upward movements showed higher variability both for first ISI and rate. (**D**) The median of the relative latencies remained rather constant at around 10ms for all velocities. (**E**) – (**H**) Velocity estimation with the maximum a posteriori (**P**) estimator on basis of absolute latencies (**E**), first ISI (**F**), spike rate (**G**) and relative latencies (**H**) with the combined data from all experiments. Perfect estimation would result in a diagonal line of pixels with 300 correct estimations each. The grey scale indicates the number of estimations. Completely random estimation would result in a grey area with a grey value of 13.64. Red crosses indicate the calculated centres of mass of the estimated velocities that were calculated for all presentations of each velocity. (**E**) Velocity reconstruction on basis of absolute latencies was close to perfect with a maximum of 205 correct estimations for a stimulus velocity of 1m/s. Centres of mass show that high velocities were slightly underestimated. (**F**) Velocity reconstruction based on the first ISI was not possible. Upward movement was overrepresented in all cases. (**G**) In the case of spike rate at least the correct direction could be reconstructed in many cases. Velocity reconstruction, however, was not possible. With relative latencies (**H**) reconstruction was inferior to the estimation on basis of absolute latencies (**E**) but clearly superior to the reconstruction based on first spike interval (**F**) and rate (**G**). Again the calculated centres of mass (red crosses) deviated mainly for high velocities. In (**I**), (**J**) (**K**) and (**L**) the reconstruction results from the best (red lines) and the worst (blue lines) trials are compared. Best results of 100% correct estimation were only observed in velocity reconstruction based on absolute latencies (**I**) followed by estimation with the relative latencies (**L**) with 18 correct estimations out of 22 velocities. Note that axes for all graphs are not linear, as the velocity steps of the stimulus differ. Data are based on 5 experiments from 5 archerfish with a total of n = 109 recorded ganglion cells.

The tuning curves of the whole population in Figures 2 A – D show the velocity dependence of the respective response component. For absolute latencies the shortest median values of 22 ms occurred at stimulus speeds around 5 and 6 m/s in both directions (Figure 2 A). This is comparable to the response latencies to light flashes of the same intensity with an average value of 21 ms (not shown). Latencies increased towards both higher and lower velocities. For the first ISI no velocity dependence of the median values could be observed (Figure 2 B). However, the response variability for all cells taken together differed between downward and upward movements. The 50% percentiles increased for the upward directions. The same was true for the spike rate (Figure 2 C). Response rate variability was smaller for downward movements compared to upward movements.

Velocity estimation with the maximum a posteriori (MAP) estimator on basis of the respective response component is shown in Figures 2 E – H. Estimations using absolute latencies yielded nearly perfect stimulus reconstruction (Figure 2 E). Errors occurred mainly in mistaking the different velocities producing similar latencies, e.g. -20 m/s and -1 m/s or the same velocities for different directions, e.g. -20 and 20 m/s. This has an influence on the centre of mass for all estimations, which is mainly shifted to lower speeds both for downward and upward movements. The best estimation by a single trial was able to correctly reconstruct all velocities (Figure 2 I, red line).

In contrast to absolute latency, the overall estimations based on first ISI (Figures 2 F & J) or rate (Figures 2 G & K) were not able to reconstruct the stimulus velocity. The best trial yielded five correct velocity estimations out of 22 for the rate (red line in Figure 2 K), but none for the first ISI (Figure 2 J). In addition, the centres of mass showed clear direction dependence in case of the rate (Figure 2 G), yet within each direction velocity could not be discriminated. Weak signs of direction discrimination were also found for the first ISI, but the centres of mass were always in the region of estimated positive direction, independent of whether the stimulus direction was upwards or downwards (Figure 2 F).

### Absolute latencies versus relative latencies

These results show that velocity estimation with absolute latencies is superior to estimation with the first ISI or the spike rate. The brain, however, has no knowledge about stimulus onset – information that is contained in absolute latency determination.

Therefore, we compared velocity estimation based on the known stimulus (absolute latencies) and the population response (relative latencies).

Since the population response results from the addition of the single responses, the population response onset latencies consequently showed a similar behaviour as the average of the single ganglion cell response latencies (the population response onset latencies are shown for the single experiments in Figures 4 K –O). Consequently, the tuning curve of the relative latencies of all ganglion cells, calculated with respect to the population response onsets, were rather constant around 10 ms (Figure 2 D). Despite the fact that the tuning curve of the relative latencies of the whole population showed no velocity dependence, the accuracy of the overall velocity reconstruction was still acceptable (Figure 2 H). Compared to estimation with absolute latencies (Figure 2 E), however, the centres of mass were more deteriorated, especially at higher velocities. The best trial still yielded 18 correct estimations out of 22 (Figure 2 L).

### Velocity tuning curve types for the different response components

The tuning curves of the whole population from Figures 2 A – D potentially mask ganglion cell subpopulations with differing tuning curves that might be important for stimulus reconstruction. Therefore, cluster analysis was applied to separate the single cells into distinct types of velocity tuning functions based on their individual tuning curves. The median values of the relevant response components for each velocity were used from each individual cell, respectively.

**Figure 3:**
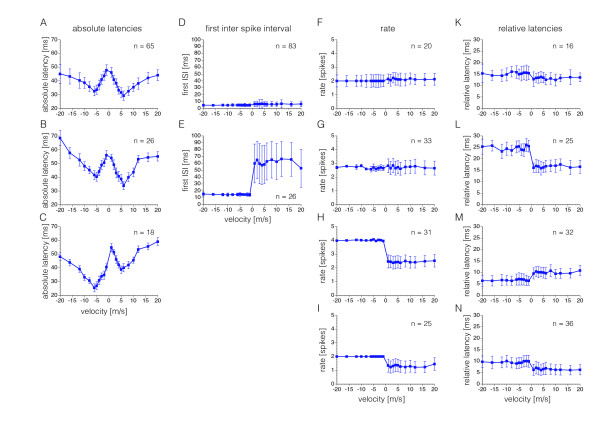
**Tuning curve types.** Cluster analysis was applied to separate the single cells into distinct types of velocity tuning functions based on their individual tuning curves. The numbers (n) indicate how many cells belong to each cluster. (**A** – **C**) One symmetrical (**A**) and two asymmetrical tuning curve types (**B** &**C**) were discriminated when using absolute latencies as response parameter. (**D** &**E**) Two types were identified for the first ISI as response parameter. None of them showed velocity tuning, however, a subpopulation (**E**) showed directional tuning. (**F** – **I**) Four types of tuning curves were clustered for rate as response parameter. Two of them showed neither velocity nor direction tuning (**F** &**G**), but one (**G**) of these two had increased inter cell variability for upward movements. This was also the case for the two remaining, directional sensitive tuning curves (**H** &**I**) that mainly differed by their response rates. (**K** – **N**) Four tuning curve types resulted when using relative latencies as response parameter. One type (**M**) with increased relative latencies for upward movement, three types with increased latencies for downward movement (**K**, **L**, **N**). These differed mainly because of different durations of relative latencies. Data are mean ± s.d.

Three W-shaped tuning curve types could be discriminated for absolute latencies (Figure 3 A – C). They were symmetrical (Figure 3 A) or asymmetrical with shortest latencies for upward movement (Figure 3 B) or with shortest latencies for downward movement (Figure 3 C).

Two tuning curves types were identified based on the first ISI (Figures 3 D & E). One had very short first ISI for all velocities (Figure 3 D) and the other type had short ISI for downward movement and long first ISI for upward movements (Figure 3 F), indicating directional tuning. The latter tuning curve type showed very high variability among cells for upward movement. Both types exhibited little variability among cells for downward movements.

Four tuning curves types were separated based on rate (Figures 3 F – I). Three of them showed a similar asymmetry in variability among cells: high variability for upward movements, very precise rates among cells for downward movements (Figures 3 H – I). Two of these tuning types (Figures 3 H & I) showed directional tuning and one had a flat tuning curve (Figure 3 G). The remaining tuning curve type is also flat, however, it exhibited similar variability among cells, both for upward and downward movements and a slightly lower average rate (Figure 3 F).

Four tuning type clusters were identified for relative latencies (Figures 3 K - N). All of them showed directional tuning: one of them with longer relative latencies for upward movement (Figure 3 M), and three with longer latencies for downward movements (Figures 3 K, L & N). The latter three types were discriminated mainly based on the duration of the relative latencies: short latencies (Figure 3 N), intermediate latencies (Figure 3 K), and long latencies (Figure 3 L).

These results show that subpopulations of ganglion cells with different tuning curves exist for the various response components. By comparison of Figure 3 with Figure 2 these results also qualitatively explain the differences in stimulus reconstruction using the different response parameters. The good velocity tuning in all three tuning curve types for absolute latencies is in accordance with the good velocity reconstruction using this parameter (Figure 2 E). For rate the directional selective tuning curves of about half of the recorded cells are in accordance with the good reconstruction of movement direction (Figure 2 G), but since no velocity tuning exists in the tuning curves, velocity could not be reconstructed. In the case of the first ISI only 26 out of 109 cells showed directional tuning, with high variability for upward movements among cells. In the reconstruction this obviously led to some degree of direction reconstruction, as indicated by the centres of mass in Figure 2 F, however, with a shift in the reconstruction to upward movement. For the case of relative latencies the tuning curves are qualitatively in good agreement with the centres of mass for velocity reconstruction shown in Figure 2 H. It remains still unclear, however, how the remaining velocity reconstruction, indicated by the diagonal in Figure 2 H, is accomplished.

### Comparison of individual experiments

**Figure 4:**
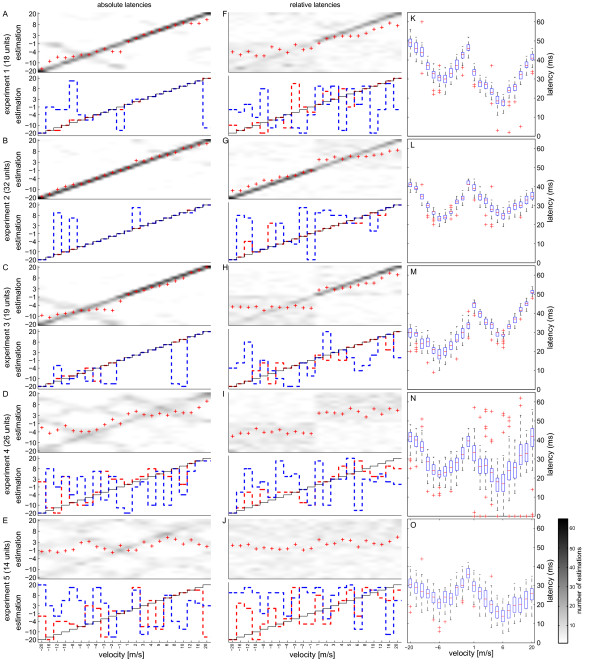
**Comparison of different experiments.** The 5 experiments showed differences in reconstruction quality based on absolute latencies (**A** to **E**). Experiments 1 - 3 (**A** - **C**) showed very good results while in experiments 4 and 5 (**D** + **E**) many errors occurred by confusing speeds with similar absolute latencies (compare to **N** and **O**) and/or opposite directions. The best trials (red lines) showed nearly perfect reconstruction in experiments 1-3 (**A** - **C**) and even the worst trials (blue lines) estimated the correct velocity far above chance level. For experiments 4 and 5 this was not the case. (**F** - **J**) Qualitatively similar results were obtained for velocity reconstruction based on relative latencies in experiments 1 - 3. However, the overall reconstruction quality slightly deteriorated. Reconstruction worsened especially for upward movements in experiment 2 (**G**) and for downward movements in experiments 1 and 3 (**F** + **H**). In experiment 4 (**I**) only direction reconstruction was possible. Velocity reconstruction with data from experiment 5 was not possible at all (**J**). (**K** – **O**) The tuning curves of the latency from stimulus onset to population response onset separated for the single experiments show higher variability in experiments 4 (**N**) and 5 (**O**), compared to experiments 1 to 3 (**K** – **L**). This indicates that the timing of the population response and therefore the single cell responses are less precise. Chance level is at 2.73 (4.55%) for each pixel in the single experiments. The number of analysed cells in each experiment is indicated on the left side.

The ability to reconstruct the different velocities on the basis of latencies differed between the individual experiments. Whereas three of the five experiments showed a very good reconstruction using latency as response parameter (Figures 4 A – C), two experiments performed significantly worse (Figures 4 D and E), independently whether reconstruction was based on absolute or relative latencies (Figures 4 D, E, I, J).

When using absolute latencies for estimation obviously some information about stimulus velocity was retained in experiments 4 and 5 (Figures 4 D and E). Most errors occurred in mistaking different velocities producing similar latencies or mistaking velocities for opposite directions. This information was gone when using relative latencies, where only estimation of the correct direction was possible in experiment 4 (Figure 4 I) and no correct estimation at all was possible in experiment 5 (Figure 4 J). The tuning curves of the population response onsets showed clear velocity dependence in all experiments (Figure 3 K - O), but the variability in experiments 4 and 5 was considerably higher. Since the population response onset is determined by the sum of the single cell responses this led us to the suggestion that precision of single cell responses might vary between experiments.

### Precision of single cell latencies

**Figure 5:**
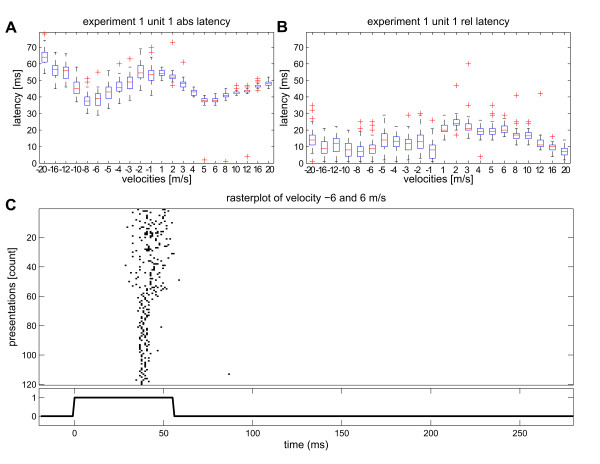
**Variability of response latency in single cells.** The latency tuning curves of many cells showed differing response variability, depending on the direction of movement, as depicted in this example from unit 1 in experiment 1. (**A**) In this example, the typical W-shaped tuning curve for absolute latencies showed increased variability for downward movements (negative velocities). For upward movements absolute latencies were more precise. (**B**) This difference in variability between downward and upward movements was retained in the tuning curve for relative latency. In addition, shallow velocity tuning was preserved for upward movements (positive velocities). (**C**) Raster plot of the same cell showing the spike responses to all stimulus presentations with velocity -6 m/s and +6 m/s. The randomly presented stimuli were sorted for this diagram. Presentations 1 to 60 indicate all downward stimuli with -6 m/s velocity, presentations 61 to 120 all upward stimuli with +6 m/s velocity. The responses to upward movement show more precise timing than responses to downward movement.

The temporal difference between population response onset and the following spike of each individual cell, respectively, is the relevant parameter for the MAP estimation based on relative latencies. Therefore, global tuning curves from all cells (Figure 2 D), and average tuning curve types from subpopulations of cells as shown in Figure 3 K - N, potentially mask important information retained in single cell tuning curves. An example is shown in Figure 5 for one cell from experiment 1. The tuning curve for absolute latencies from this cell is symmetrical and belongs to the tuning type shown in Figure 3 A. However, the individual tuning curve shows considerable differences in the variability of the absolute latency between upward and downward velocities, not retained in the average tuning curve from Figure 3 A. A raster plot of the responses of this cell to all presentations of the stimuli with velocities of -6 m/s and +6 m/s illustrates this differing behaviour for upward and downward movement (Figure 5 C). This difference in variability is also visible for the tuning curve based on relative latencies (Figure 5 B), which belongs to the tuning curve type shown in Figure 3 M. In addition, this cell still exhibits shallow velocity tuning for upward movements, the region of the tuning curve with low variability.

**Figure 6:**
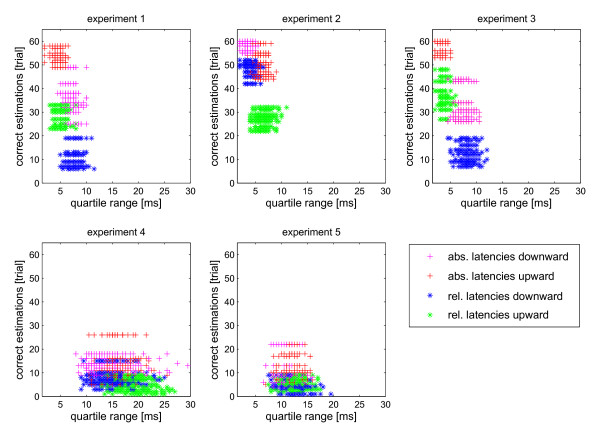
**Correlation of correct estimation with variability of response latency.** The number of correct estimations was plotted against the variability of the response latency of each cell, quantified by the median of the latency quartile ranges. Data are separately shown for upward and downward movements, both for absolute and relative latencies. Each of the five experiments was analyzed separately. In experiments 1 – 3 the number of correct estimations for upward and downward directions (indicated by different colours) correlated with the quartile range when considering estimations based on absolute and relative latencies separately. Small quartile ranges correlated with better estimation and vice versa. However, the number of correct estimations for the same direction was always lower when based on relative latencies. Experiments 4 and 5 showed high variability in response latencies and very low estimation quality.

This observation suggested that the estimation quality might correlate with the precision of the latencies of individual cells, which in turn might be different for upward and downward movements. With high precision latencies even a shallow velocity dependence will contribute to correct velocity reconstruction. Therefore, we determined the quartile ranges as a measure of precision for each velocity and for each cell separately and plotted these values against the number of correct estimations for each cell. This was done for absolute and relative latencies, and the results are shown in Figure 6, separately for the different experiments 1 to 5. In the experiments 1 – 3, with good reconstruction, quartile ranges were in most cases below 10 ms. In addition, the number of correct estimations correlated with the quartile range. A smaller quartile range correlated with a higher number of correct estimations, a larger quartile range with a lower number of correct estimations. This was independent whether absolute or relative latencies were used or whether upward or downward movement was applied. The difference between absolute and relative latencies was, however, that even for similar quartile ranges the number of correct estimations was always smaller when based on relative latencies (see for instance Figure 6; experiment 3). In contrast to experiments 1 to 3, quartile ranges were mostly above 10 ms in experiments 4 and 5. This correlated with low numbers of correct estimations.

**Figure 7:**
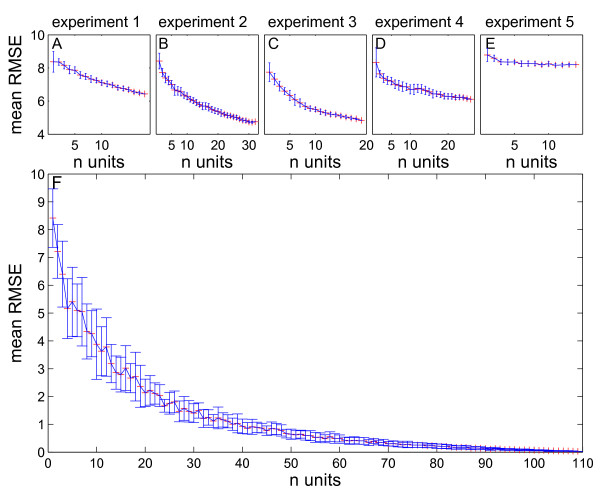
**Reconstruction error for increasing number of ganglion cells.** (**A** – **E**) The mean of the root mean square error (RMSE) for stimulus reconstruction with relative latencies was calculated for each experiment with random choice of increasing number of units. The reconstruction error decreased monotonically in all experiments but experiment 5, where error remained high (E). Especially experiment 2 with the highest number of recorded cells (n = 32) yielded a very low error of 4.72 m/s. (**F**) When calculating the mean RMSE with increasing number of all 109 ganglion cells from all 5 experiments, the error approached zero. n = 18 in (A); n = 32 in (B); n = 19 in (C); n = 26 in (E); n = 14 in (E), total n = 109 (F).

When iteratively increasing the number of ganglion cells used for estimation, the calculated mean of the root mean square error (RMSE) for stimulus reconstruction with relative latencies decreased for all individual experiments (Figures 7 A-E). Experiment two (Figure 7 B) with the largest number of recorded cells (n = 32) performed best with a mean RMSE of 4.72 m/s. In experiment 5 the mean RMSE stayed almost constant at a high level (Figure 7 E). When estimating the stimulus velocity with all 109 units of the combined experiments, the mean RMSE decreased to 0.028 m/s with a parallel decreasing standard deviation.

## Discussion

Archerfish ganglion cells are potentially able to contribute to the high speed calculation of a prey impact point on the water surface by coding different speeds and directions with precisely timed spiking. Ganglion cell responses show a very low noise level and response rate. With latencies down to 12 ms after the onset of a moving stimulus some responses are faster than recorded latencies to light flashes of the same intensity and to ganglion cell latencies of other species to comparable moving stimuli e.g. 100 ms in turtle or 50 ms in rabbit and salamander [21,22].

Precisely timed and directed motor reactions in archerfish can occur at latencies down to 40ms after presentation of falling prey [2]. It is likely that this motor reaction is triggered by the Mauthner cells [3] because these are directly electrically coupled to motorneurons over descending interneurons and hence permit very fast signal transmission of only a few milliseconds [23]. Mauthner cells are also involved in other visually evoked behaviour like looming, where latencies of 142 ms have been observed, and feeding [24,25]. Compared to sound induced responses with latencies of 10 to 15 ms [24], these longer latencies in these visually evoked behaviours reflect the comparably slow phototransduction and probably the increased amount of pre-processing, that might be involved to calculate the transformation of size and velocity and the potential time to collision with an approaching object, e.g. a predator. Similar stimulus parameters must be estimated by the archerfish nervous system to calculate the point of impact of the prey on the water surface, but the behavioural response takes place on a much shorter timescale, suggesting a time-optimized neural code.

In this study the best velocity reconstruction could be achieved on the basis of absolute latencies. In the case of the reconstruction with rates performance was overall very poor, and in the case of reconstruction with first ISI only some reconstruction of stimulus direction was possible.

Therefore, archerfish ganglion cells might encode information about stimulus speed and direction through the precise timing of first spikes. Since the brain has no knowledge about the stimulus onset, latency can only serve as a coding parameter when information about the stimulus onset is represented by the response of a cell population. Precision of the single cell responses with respect to the population response onset becomes then an important parameter too. When taking the population responses of all recorded cells into account estimation of the 22 velocities with relative latencies was possible far above chance level and above the level achievable by estimating velocities on the basis of rates or first inter spike intervals. The results clearly depend on the amount of cells involved in the estimation and on the precision of the recorded ganglion cell response latencies.

Comparison of our single experiments shows that under favourable conditions (Experiment 2) on the average 63% (38 out of 60) correct estimation could be obtained, compared to a chance rate of 4.5%. The fact that single cell precision was less precise in experiments 4 and 5 supports the notion that spiking precision is very critical for this task. It is unclear why less precise cells were recorded in experiments 4 and 5. It could of course be that the quality of the experiments was inferior in experiments 4 and 5, but this seems unlikely to us since we recorded more cells in experiment 4 than in experiments 1 and 3. It could as well be that by chance more cells with intrinsically less precise responses were recorded in experiments 4 and 5. We took care to align the retina dorso-ventral in our experiments. However, we had no good control how far dorsal or ventral the recordings took place. Since the archerfish retina is not homogeneous, with a ventral region of increased photoreceptor and ganglion cell density [26] it could be that we recorded more cells from one region or the other region in the different experiments.

In a competition model, based on directionally selective ganglion cells in archerfish, simulated reaction times for the decision to move to the left or to the right were in good accordance with behavioural data [27]. In this study directionality was based on rate, leading to about 42% directionally tuned cells, which is similar to our number of 51% directionally tuned cells when using rate as response parameter (Figure 3). Therefore rate might be an additional possibility for making the decision to move left or right. However, the authors also showed that directional selectivity based on rate is invariant to changes in velocity, the same result we obtained (Figure 2 G). The task for the archerfish is, however, to calculate the impact point of shot down prey before it reaches the water surface [2,3]. For doing this, knowledge of the speed of the prey in addition to its direction is indispensable. From our results only latency can serve as response parameter for this task.

Generally, population response onset is a good indicator of stimulus changes, even in cases with ongoing spike activity [12]. Therefore, it seems reasonable to use this as a time reference for relative latency determination that could be used by the nervous system. The definition of population response onset is, however, arbitrary. In the case of archerfish, with low spontaneous spike rate under our experimental conditions, relative latencies could also be calculated with respect to the first spike within a cell population. This is basically the same as lowering the threshold of the population response onset to one spike. For comparison, we therefore also estimated the stimulus velocities based on relative latencies with reference to the first spike for each stimulus presentation. Velocity estimation was, in this case, comparable to estimation based on absolute latencies (Additional file 1: Figure S1).

We do not know whether the task of velocity estimation is based on the activity of all cells in a certain retinal region or the activity of a subset of cells responding very precisely. It is also not clear whether the brain uses the population response as reference signal, and if yes whether it uses the population response from all retinal ganglion cells or from a subset of very precisely responding cells. Even the spike of the fastest responding cell could serve as a reference signal as long as the ongoing activity is low. The question is, however, how the ganglion cell activity looks like under natural viewing conditions and how reliable the different reference signal then are.

## Conclusions

The results presented in this study show that reliable and fast velocity reconstruction can be obtained with latency as response parameter of retinal ganglion cells. This is true for absolute as well as relative latencies. The latency available for the archerfish is composed of the population response latency (20 – 30 ms at the optimal velocities) plus the relative latency (around 10 ms) and, therefore, in the range of 30 – 40 ms. Since the transmission from the optic nerve to a Mauthner cell response takes only about 4 ms [28], this is in good agreement with the fastest reaction times of 40 – 45 ms measured in behavioural experiments [2,27].

## Methods

All animal experiments were performed in compliance with the guidelines for the welfare of experimental animals issued by the European Communities Council Directive of 24 November 1986 (86 609 EEC) and the laws of the Federal Government of Germany (Tierschutzgesetz; BGBl. I S. 1206, 1313 and BGBl. I S. 1934). Institutional approval was obtained by the ethical committee of the University of Oldenburg.

### Electrophysiology

Extracellular multi-electrode recordings from ganglion cells of the archerfish (*Toxotes chatareus*) retina were performed using a ten by ten silicon array (Blackrock Microsystems; Salt Lake City, UT, USA) with an inter-electrode distance of 400 μm. Animals were decapitated and the retina was removed keeping the pigment epithelium attached. The flattened retina/pigment epithelium preparation was then placed into the translucent recording chamber. In the archerfish the pecten and different coloration of the dorsal/ventral retina can serve as landmarks. Incisions, that are necessary to flatten the retina, were made along the temporal/nasal line that served then as landmarks to keep track of the orientation. In the recording chamber the retina was oriented in such a way that the final stimulation with the moving grating was dorso-ventral. During the experiment the preparation was constantly superfused with oxygenated ringer solution (120mM NaCl, 5mM KCl, 2mM CaCl_2_, 2mM MgCl_2_, 10mM glucose, 22mM NaHCO_3_, bubbled with 95% O_2_ -5% CO_2_ ; pH7.4). The temperature within the recording chamber was held constant at 20°C by using a temperature controlled, translucent heating chamber underneath the preparation. Ganglion cell activity was pre-amplified, sampled and stored by a 128-channel Cerebus neural signal acquisition system (Blackrock Microsystems; Salt Lake City, UT, USA).

After an experiment spikes were sorted with the Plexon Offline Sorter, Version 2.8.8 (Plexon Neurotechnology Research Systems, Dallas, Tx, USA). The supervised *k*-means clustering algorithm was used to cluster spike waveforms on the basis of principle component analysis. All further analysis with the resulting time stamps from the sorting procedure was done in MATLAB, Version 7.11.0.584 (The MathWorks Inc., Natick, Mass, USA).

### Light stimulation

Light stimulation was realized using a white high-power LED (LXHL-FW6C; Luxeon, San Jose, CA), with broad emission spectrum as light source. Full field light flashes (12 mW/m^2^ on the retina) of 50 ms duration (1.54 Hz) were used to search for ganglion cell responses while penetrating the preparation. When enough ganglion cells responded to the search flashes, light stimulation was switched to a regular grating used for stimulation with different velocities. The grating was composed of black and white bars, each with a width of 400 μm. About 80% of the cells that responded to the search flashes responded also to the movement stimulation. A few cells that responded to movement did not respond to the search flashes. A total of 109 cells responding to movement were recorded in five retinae from five different archerfish.

The grating was generated by constantly projecting the image of a photographic slide onto the retina. The slide was composed of non-transparent and transparent stripes. Light intensity on the illuminated parts of the retina was the same as above. The edges of the stripes were initially aligned with the electrode rows (Figure 1 G). The image of the slide could then be moved with various speeds perpendicular to the bar orientation (direction of bar movement dorso-ventral on the retina) by using an x-y miniature mirror system (Datronik, Rastede, Germany) connected to a stimulus computer. The stimulus computer was synchronized with the data acquisition computer for exact stimulus on- and offset determination. Each movement stopped after 400 μm, when edges of the bars reached the neighbouring electrode rows. Movement direction changed after every second movement step. This ensured that the grating reversed in half of the stimuli. The third movement started into the opposite direction. Eleven different speeds (1 m/s, 2 m/s, 3 m/s, 4 m/s, 5 m/s, 6 m/s, 8 m/s, 10 m/s, 12 m/s, 16 m/s, 20 m/s) were used, which were calculated according to the speed distribution of natural objects at approximately 30 cm distance [2,29]. These speeds correspond to retinal stimulus velocities of 1.21 mm/s; 2,42 mm/s; 3,63 mm/s; 4,84 mm/s; 6,05 mm/s; 7,25 mm/s; 9,67 mm/s; 12,09 mm/s; 14,50 mm/s; 19,34 mm/s; 24,18 mm/s. The different velocities were applied in random order. Since the distance of movement was constant, stimulus duration varied according to movement speed. In the text, the two different directions were named downwards (movement into dorsal direction on the retina) and upwards (movement into ventral direction on the retina), in order to refer to the movement direction of potential prey in the environment. In the figures downwards and upwards were referred to as negative and positive velocities, respectively.

Each of the 22 velocities ( = speed and direction) was presented 60 times in random order, yielding 1320 stimulus presentations per experiment. Presentations were separated by a break (600 ms minus the varying time of movement; see Figure 1) from the next stimulus. Since the grating reversed every second movement step the illuminated and dark bars were on either side of the electrodes at the start of 30 trials, respectively. We tested whether this grating reversal had an effect on the tuning curves by subdividing the data for the two grating phases. The result is shown in Additional file 2: Figure S2 for the data based on absolute latency. Since we found no difference in the velocity tuning curves between the reversed and non-reversed grating we put all data together for further analysis.

### Cluster analysis of tuning curves

In order to identify different tuning curve types, individual cells were classified according to the form of their individual tuning curves. We used the k-means clustering algorithm implemented in JMP 7.01 (SAS Institute Inc., Cary, NC, USA). The feature vector was 22-dimensional, consisting of the median values of the response component under study (absolute and relative latency, first ISI, rate) at each velocity, respectively. The k-means algorithm demands a predefined number of clusters. For obtaining this number we first visually inspected 2- and 3-dimensional plots of the two and three most important principal components and subjectively decided how many clusters were predefined. In addition, the resulting clustering was inspected in diagrams showing the overlay of the respective tuning curves. Finally, the average distance of all tuning curve vectors of a given cluster to the cluster centre was calculated. The resulting numbers were then compared to the average distances for clustering with one cluster more or one cluster less. If the average distance was smallest this cluster number was accepted.

### Stimulus reconstruction

The stimulus parameters speed and direction (= velocity) were reconstructed by analyzing three parameters of the ganglion cell responses: latency of the first spike; first inter-spike interval (ISI); rate within a 100 ms window after stimulus onset. Latency of the first spike was determined for two cases: latency with respect to stimulus onset (absolute latency) and latency with respect to population response onset (relative latency). For determination of the population response onset the spike trains of all cells were added and the resulting data smoothed by a moving average. Then population response onset was arbitrarily defined for each stimulus presentation as the time point where the population response reached 2.5 times the standard deviation [30].

Stimulus reconstruction was done using a maximum a posteriori (MAP) estimator following the procedure described previously [12]. The MAP estimator includes the distribution of the known stimulus parameters. The aim is to identify the most probable stimulus θ given the observed data X by maximizing the posterior distribution p (θ|X): θ_MAP_(x) = arg max_θ_ p (θ|X). Applying Bayes´theorem yields:

$$\;\theta_{\mathrm{MAP}}\left(x\right)=\arg\max_{\theta }f(X|\theta )s(\theta )$$

with f (X|θ) being the likelihood function, the probability of observed data X given the stimulus θ. s (θ) is the prior distribution of the stimuli.

For the computation the trials, consisting of one presentation of each velocity (yielding in total 60 trials each with 22 velocities), were separated into training sets of 59 trials and one trial used for testing. Each trial was used as test set using a “jackknife” procedure. This procedure minimizes overfitting effects. For each of the trials used for training we calculated the empirical distribution and/or the Bernoulli distribution for each recorded unit. The response to each presented velocity of the trial used for testing was then compared to one of the distribution functions, for the respective response parameter, to calculate the probability that the particular stimulus was presented. When a spike occurred within 100 ms after stimulus onset, the empirical distribution was used, whereas the Bernoulli distribution was used for responses later than 100 ms after stimulus onset. The product of all probabilities from all units was then calculated and multiplied with the prior, the frequency distribution of the different velocities. The maximum probability is then taken as the most likely stimulus velocity.

In our case the priori distribution is uniform with an equal probability for each velocity. In total there are *K* trials and the number of all units is *N*. Let *T* be the random variable for the stimulus occurrence.

The likelihood function of the random variable *X* for each observation *x*_*i*_ of unit *i* in trial *k* was estimated from the training dataset for all 100 ms time intervals $T_{\theta }$ where stimulation *θ* occurred. *M*_*t*_ is defined as the set of all units that elicit spikes within the specified time interval $t\in T_{\theta }$.

For latency and first interspike interval it is

$$\;\begin{array}{cc} f_{X|T=\theta }^{k}\left(x\right)=\prod_{i\in M_{t}}f_{X_{i}|T=\theta }^{k}\left(x_{i}\right), & x_{i}\in \chi =[0\mathrm{ms},100\mathrm{ms}] \end{array}$$

For spike count *x*_*i*_ was defined as

$$\;x_{i}\in X=[0\mathrm{spikes},20\mathrm{spikes}]$$

In cases were no spikes where detected within 100 ms after stimulus onset a Bernoulli distribution was used instead, which only takes into account whether or not a spike occurred later.

$$\;b_{t,i}^{k}=\{\begin{array}{ll} 1 & \text{if}\;one\;or\;more\;spikes\;were\;evoked\;for\;unit\;i\in \{1,\ldots ,N\}\\ & \text{in}\;the\;time\;window\;t\in T_{\theta }\;\text{in}\;trial\;k\in \{1,\ldots ,K\}\\ 0 & \text{otherwise} \end{array}$$

Under the assumption that variates are independent, the likelihood function results as a product of Bernoulli-densities:

$$\;\begin{array}{cc} f_{Y|T=\theta }^{k}\left(y\right)=\prod_{i=1}^{N}g_{i}^{k}{\left(\theta \right)}^{y_{i}}\cdot {\left(1-g_{i}^{k}(\theta )\right)}^{1-y_{i}}, & y_{i}\in Y=\{0,1\} \end{array}$$

with the probability $g_{i}^{k}$ which was estimated from the training dataset (excluding the test set) and all time intervals $t\in T_{\theta }$ where stimulation *θ* occurred.

$$\;g_{i}^{k}\left(\theta \right)=\frac{1}{\#T_{\theta }}\frac{1}{K-1}\sum_{t\in T_{\theta }}\underset{k*\neq k}{\sum_{k*\in \{1,\ldots ,K\}}}b_{t,i}^{k*}$$

The combined likelihood estimation can then be described as follows:

$$\;\begin{array}{cc} f_{Z_{i}|T=\theta }^{k}(z_{i})=f_{(X_{i},Y_{i})|T=\theta }^{k}(x_{i},y_{i})= & \left\{\begin{array}{ll} f_{Y_{i}|T=\theta }^{k}\left(0\right), & y_{i}=0\\ f_{X_{i}|T=\theta }^{k}\left(x_{i}\right), & y_{i}=1 \end{array}\right) \end{array}$$

The likelihood function for all *N* units is

$$\;f_{Z|T=\theta }^{k}\left(z\right)=\prod_{i=1}^{N}f_{Z_{i}|T=\theta }^{k}\left(z_{i}\right)$$

Additionally we calculated a centre of mass for all estimations for one velocity to determine an overall estimation tendency (e.g. to get an indication for the overall direction preference).

The centre of mass *R* was defined as:

$$\;R=\frac{1}{V}\sum v_{i}\cdot c_{i}$$

with the locations *c* weighted by their values *v* and *V* as the sum of all values for each estimation *i*.

For the determination of the quality of the estimation the root mean square error (RMSE) was calculated. The best estimation was determined as the trial with the minimum overall deviation from the actual stimulus. To analyze whether the quality of the estimation correlates with the amount of units recorded we calculated the root mean square error while iteratively increasing the number of units. The RMSE was calculated for all trials *i* for the actual stimulus *y* and the estimation *x* in all trials (*n* = 60):

$$\;RMSE=\sqrt{\frac{\sum_{i=1}^{n}{\left(x_{i}-y_{i}\right)}^{2}}{n}}$$

This was done ten times by randomly picking the number of units from the dataset, and the mean of these ten values was defined as the error measure.

## Competing interests

The authors declare that they have no competing interests.

## Authors' contributions

V.K. performed the experiments, data analysis, programming of evaluation routines and contributed to the article. F.K. contributed to the data analysis, evaluation routines and article. M.T.A. built the setup and the temperature control system and assisted with experimental expertise. J.A. supervised the project, assisted in experiments and contributed to the article. All authors’ read and approved the final manuscript.

## Supplementary Material

Additional file 1 **Figure S1.** Velocity estimation with reference to first spike. For comparison with the estimations based on relative and absolute latencies, shown in the main text, we also performed estimations using the first spike in response to each stimulus presentation as temporal reference point. (A) The median of the tuning curve from the whole population for latencies with reference to the first spike remained rather constant between 10ms and 15 for all velocities. This is similar to the tuning curve with relative latencies with respect to population response onset, as shown in **Figure**2**D**. (B) Estimation quality, however, was comparable to estimation quality based on absolute latencies, as shown in **Figure**2**E**. Especially the centres of mass improved considerably, compared to estimation based on relative latencies (compare to **Figure**2**H**). (C) The reconstruction results from the best (red line) and the worst (blue line) trials are also comparable to estimation based on absolute latencies (**Figure**2**I**). See figure legend for **Figure**2 for details. Click here for file

Additional file 2 **Figure S2.** Grating reversal had no effect on latency tuning curves. We tested whether the grating reversal had an effect on the tuning curves based on absolute latencies by subdividing the data for the two grating phases. The tuning curves for each grating phase are shown in (A) and (B), respectively, for each single cell (coloured points). Crosses indicate median values from all cells. No difference was visible. Click here for file
