# Supplementary figures and images for: High speed coding for velocity by archerfish retinal ganglion cells
Source: BMC Neurosci. 2012 Jun 18;13:69. doi: 10.1186/1471-2202-13-69 (PMC3508827; doi:10.1186/1471-2202-13-69)

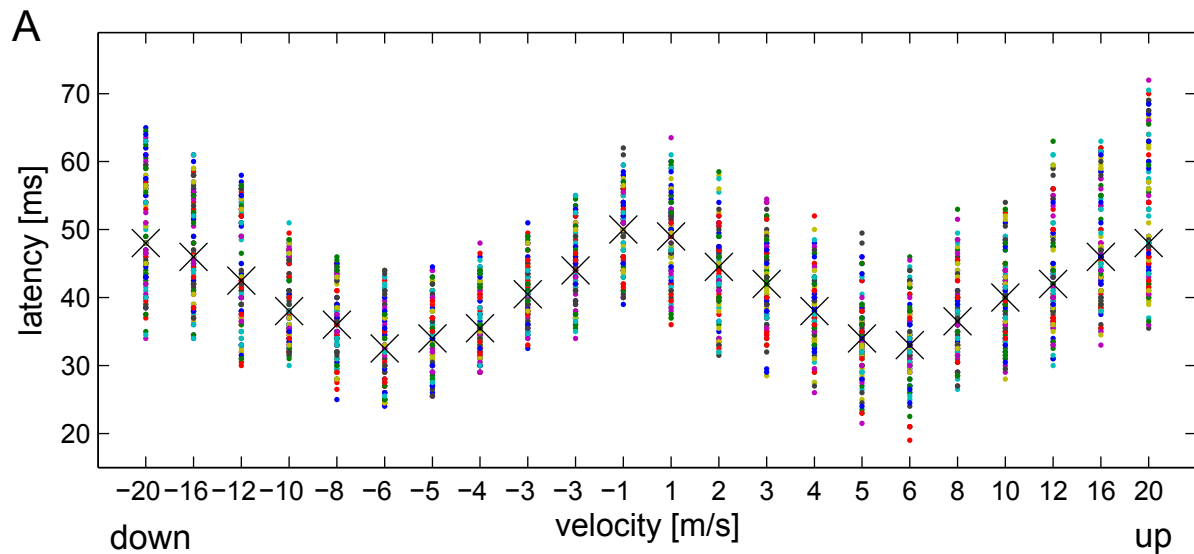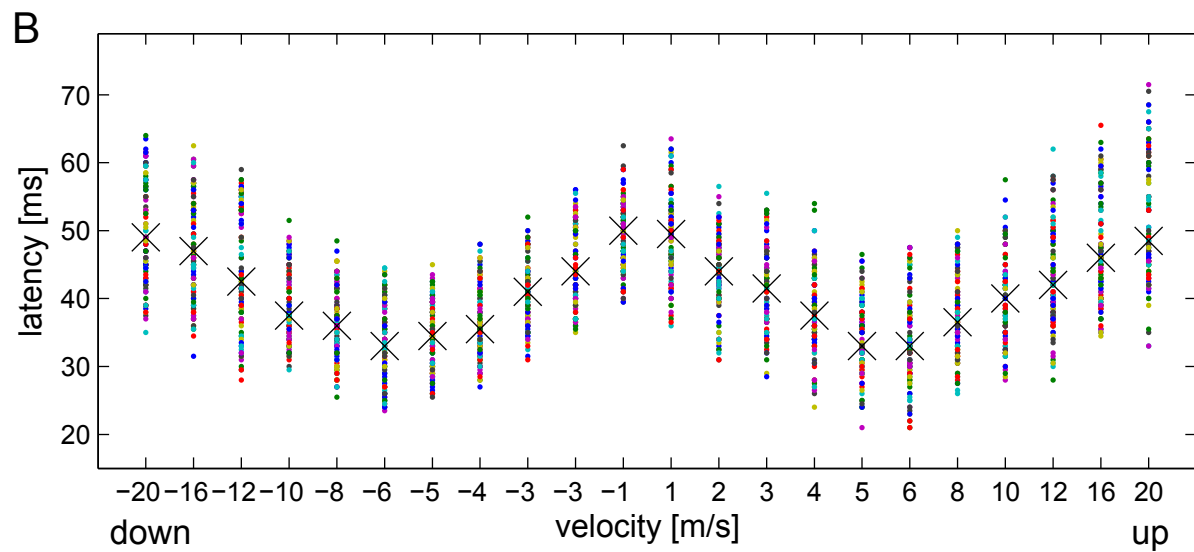

Supplement: Additional file 1 — Figure S1. Velocity estimation with reference to first spike. For comparison with the estimations based on relative and absolute latencies, shown in the main text, we also performed estimations using the first spike in response to each stimulus presentation as temporal reference point. (A) The median of the tuning curve from the whole population for latencies with reference to the first spike remained rather constant between 10ms and 15 for all velocities. This is similar to the tuning curve with relative latencies with respect to population response onset, as shown in Figure2D. (B) Estimation quality, however, was comparable to estimation quality based on absolute latencies, as shown in Figure2E. Especially the centres of mass improved considerably, compared to estimation based on relative latencies (compare to Figure2H). (C) The reconstruction results from the best (red line) and the worst (blue line) trials are also comparable to estimation based on absolute latencies (Figure2I). See figure legend for Figure2 for details. [file 1471-2202-13-69-S1.pdf]

# **A** relative latencies with reference to first spike

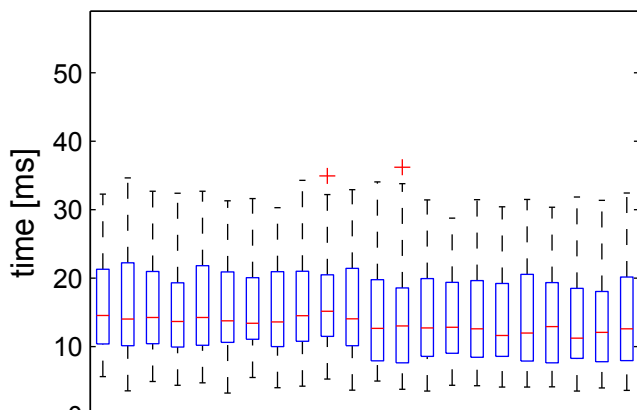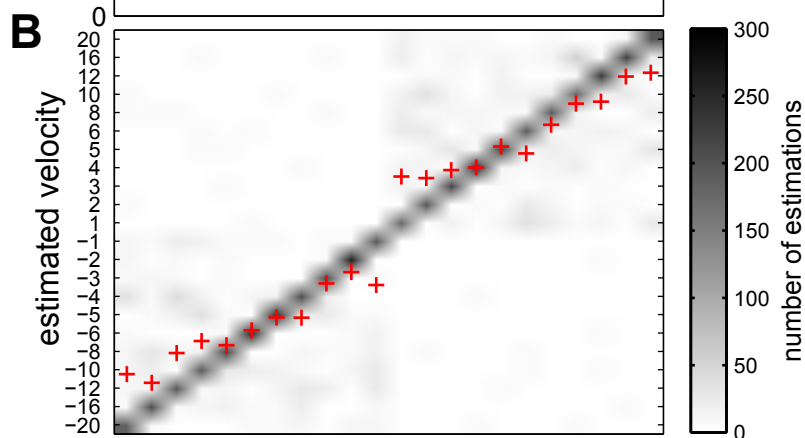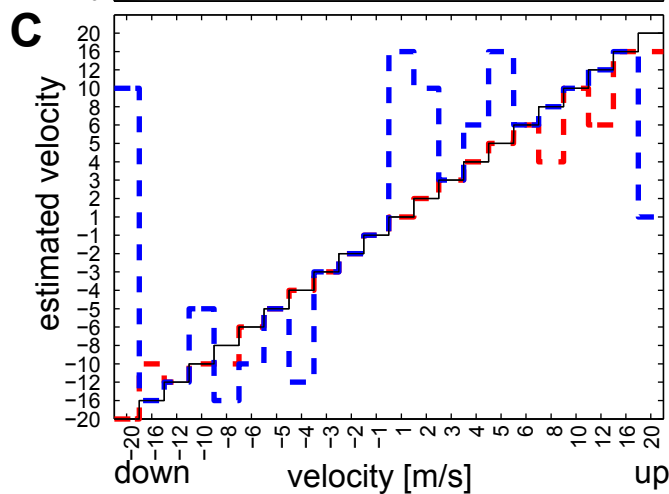

Supplement: Additional file 2 — Figure S2. Grating reversal had no effect on latency tuning curves. We tested whether the grating reversal had an effect on the tuning curves based on absolute latencies by subdividing the data for the two grating phases. The tuning curves for each grating phase are shown in (A) and (B), respectively, for each single cell (coloured points). Crosses indicate median values from all cells. No difference was visible. [file 1471-2202-13-69-S2.pdf]
